# Vital Functions Contribute to the Spread of Extracellular Fluids in the Brain: Comparison Between Life and Death

**DOI:** 10.3389/fnagi.2020.00015

**Published:** 2020-02-11

**Authors:** Alina Piotrowska, Karsten Winter, Roxana O. Carare, Ingo Bechmann

**Affiliations:** ^1^Institute of Anatomy, Leipzig University, Leipzig, Germany; ^2^Faculty of Medicine, University of Southampton, Southampton, United Kingdom

**Keywords:** clearance of the brain, blood-brain-barrier, cervical lymph nodes, Virchow-Robin space, glymphatic system

## Abstract

Vascular pulsations, contractions of vascular smooth muscle cells and breathing have been reported to foster movement and clearance of interstitial and cerebrospinal fluids from the brain. The aim of this study was to estimate the contribution of these vital functions. We compared the spread of an injected hydrophilic tracer (Fluoro-Emerald, a 10 kDa fluorescein-coupled dextran amine) in the brains of live anesthetized and sacrificed rats at 30 and 90 min after injection. To determine the overall pattern of distribution of tracers, we created 3D-reconstructions of the horizontal transections of the whole brain. Immunofluorescence staining with laminin and collagen IV was performed to determine the pattern of distribution of tracer in relation to the cerebrovascular basement membranes. We found that diffusion was widely restricted to the periventricular region in sacrificed rats with no spread to the contralateral hemisphere, while the bulk flow occurred along the vasculature and reached the surface and the contralateral hemisphere as soon as 30 min after injection in live anesthetized animals. The tracer appeared to be localized along the vascular basement membranes and along fiber tracts as reported previously. Thus, our data indicate that vital functions are essential for the remote movement of extracellular fluids within the cerebral parenchyma.

## Introduction

The immune responses of the brain are restricted by the presence of a blood-brain barrier and the lack of traditional lymphatic vessels (Bechmann et al., 2007; Galea et al., 2007; Abbott et al., 2018), but homeostasis of extracellular fluids is crucial for synaptic transmission (Ivens et al., 2007). While it is clear that IFS and soluble antigens drain into cervical lymph nodes (Schwalbe, 1869; el-Gindi et al., 1973; Rosenberg et al., 1980; Cserr and Knopf, 1992; Kida et al., 1993; de Vos et al., 2002; Koh et al., 2005; Goldmann et al., 2006; Kaminski et al., 2012; Locatelli et al., 2012; Ma et al., 2017, 2019), the driving forces for extracellular movements and the respective pathways have only been identified and are still under debate (Cserr et al., 1977; Rosenberg et al., 1980; Abbott et al., 2018; Gakuba et al., 2018; Plog and Nedergaard, 2018; Ma et al., 2019). One highly discussed pathway is the “glymphatic system” consisting of a convective influx system of the CSF into the brain (Iliff et al., 2012; Abbott et al., 2018). According to the “glymphatic” hypothesis CSF and ISF exchange takes place from the subarachnoid space via periarterial Robin-Virchow spaces, through the brain parenchyma toward perivenous spaces (Iliff and Nedergaard, 2013; Iliff et al., 2013; Nedergaard, 2013). Tracers in the CSF are thought to enter the parenchyma as convective flow along Virchow-Robin spaces of arteries, where AQP4 expressed on astrocytic endfeet of the *glia limitans* perivascularis is supposed to play a major role (Iliff et al., 2012), and return to the CSF via the walls of veins but this process has been extensively debated (Hladky and Barrand, 2014; Asgari et al., 2016; Bakker et al., 2016; Holter et al., 2017; Abbott et al., 2018; Faghih and Sharp, 2018; Gakuba et al., 2018; Ma et al., 2019). The anatomical route of entry of the CSF into the brain is along the basement membranes of the cerebral arterioles (pial-glial basement membranes) and the efflux of ISF is along the basement membranes surrounding arterial smooth muscle cells, the IPAD pathway (Albargothy et al., 2018). The convective influx/glymphatic network can be seen as a low resistance network and is believed to play a major role in spreading of large molecules such as 150 kDa IgG (Pizzo et al., 2018) and smaller molecules such as 40kDa peroxidase (Cserr and Ostrach, 1974; Cserr et al., 1977). CSF including different waste products experimentally tested using intracisternally injected tracers drain to cervical lymph nodes via several pathways along cranial nerves as well as spinal cord roots (Bradbury et al., 1981; McComb, 1983; Kida et al., 1993; Koh et al., 2005; Ma et al., 2017). Direct drainage to the blood via arachnoid villi has been postulated for a long time, but its contribution to CSF outflow is currently under discussion (Ma et al., 2017). As driving forces, vascular pulsations (Harrison et al., 2018) have been shown to contribute to perivascular movement within the perivascular channel system, as unilateral ligation of one carotid artery reduced vascular pulsatility and spread of a tracer (Iliff et al., 2013; Asgari et al., 2016). Moreover, sleep (Di Rocco et al., 1975; Nilsson et al., 1992; Xie et al., 2013), breathing (Dreha-Kulaczewski et al., 2015), anesthesia and death (Ma et al., 2019) have also been accounted for the movement and clearance of intrathecal fluids, but direct visualization of the latter is still missing. Commonly used imaging techniques such as MRI (Harrison et al., 2018) and CT (Murtha et al., 2014) scans offer a low resolution compared to microscopic studies whereas most microscopic approach lack a 3D view with a satisfactory depth. The aim of this study was to visualize the net effect of vital functions on the movement and distribution of extracellular fluids within the brain after intraparenchymal injections. To this end, we monitored bulk flow of soluble Fluoro-Emerald of 10.000 molecular weight at 30 and 90 min after intraparenchymal injection in living compared to sacrificed rats.

## Materials and Methods

### Animals

Twenty-four, 3-month-old Sprague Dawley rats were used in this study. Animal husbandry was performed in the animal facilities of the Faculty of Medicine, University of Leipzig according to European (Council Directive 86/609/EEC) and German (“Tierschutzgesetz”) guidelines for the welfare of experimental animals and approved by the local authorities (Landesdirektion Sachsen; TV03/13). Rats were housed in the central breeding facility of the Medical Faculty of the University of Leipzig under standard conditions with food and water *ad libitum*.

### Stereotactic Tracer Application

**FIGURE 1:**
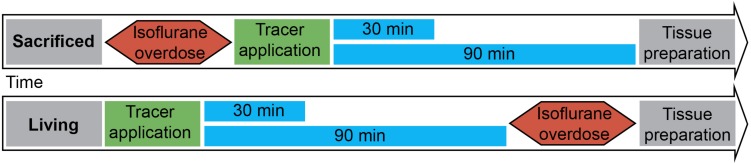
Experimental setup: 3 μl of tracer was injected into the entorhinal cortex of sacrificed and living rats over 5 min. Body temperature was constantly monitored and kept steady until brains and lymph nodes were removed after 30 or 90 min. (*n* = 7 for 30 min per group, *n* = 3 for 90 min per group).

Figure 1 shows the experimental setup. Animals were split into two groups. The first group was euthanized with an overdose of isoflurane, before stereotactic surgery. Consequently a 15% concentration of isoflurane was infused in a cylindrical glass box. The animal was placed in the box until it stopped breathing. Isoflurane exposure continued for one more minute subsequently. Death was verified manually by missing cardiac pulsation and missing blood flow during the following surgery. The injection was performed 20 min (±2 min) after death depending on the quickness of the surgical procedure described below. Animals of the second group were anesthetized with 100 mg/kg ketamine and 10 mg/kg xylazine diluted 50% in NaCl 0.9% injected intraperitoneally. All animals were put into a stereotactic frame (Narishige – Type ST-7 No106002, Tokyo, Japan) and received an injection of 3 μl of 10 mM FlouroEmerald using a 26 gauge Hamilton syringe (SYR 10 μL, #701, ga26s/51mm/pst2, P/N: 80300/00, WO 1002554, Hamilton company, Reno, Nevada). The surgery was performed as reported previously (Bechmann and Nitsch, 1997). Briefly, eyes were protected and after exposing the skull, a burr hole was drilled at antero-posterior (AP) 1, 3 mm, lateral (L) 4.5 mm from Lambda, and the syringe inserted vertically (V) for 5.5 mm, aiming for the entorhinal cortex. The tracer was applied over 5 min at a rate of 0.6 μl/min, the needle was then removed after 1 min to prevent backflow. Injection rate and volume were chosen accordingly to prevent raise in ICP (Yang et al., 2013). After the injection, the hole was sealed using bone wax (Ethicon, Johnson and Johnson International) to avert artifacts from ICP loss. In both groups, body temperature was measured using a rectal probe and adjusted to 37°C using a heat pad (TR-200, FST) on which the animals were placed. Animals of the second group were sacrificed with an overdose of isoflurane at 30 and 90 min after tracer injection.

### Tissue Preparation

The rats were dissected either 30 or 90 min after the injection, the brain was immersion fixed in 4% PFA and 1% GAD, since transcardial perfusion was impossible to perform due to coagulation in the sacrificed animals. *N* = 4 animals received transcardial perfusion and were therefore excluded from the final analysis. Lymph nodes were dissected and divided into four groups according to Takahashi and Patrick (1987): right and left DCLN, SCLN, and ILN, and also immersion fixed in 4% PFA. After 24 h the brains of *n* = 20 animals were cut horizontally into 70 μm thick slices using a vibratome (Leica VT1200). The slices were stored in 0.09% sodium azide until they were washed and stained with DAPI for 5 min. Consecutive slices of the whole brain were prepared for *n* = 10 animals: *n* = 6 for 90 min PI (*n* = 3 for sacrificed (S90) and *n* = 3 for living (L90) animals) as well as *n* = 4 for 30 min PI (*n* = 2 for sacrificed (S30) and *n* = 2 for living (L30) animals). For *n* = 10 animals at time point 30 min (*n* = 5 sacrificed and *n* = 5 living) several slices were stored in the same well and the brains could therefore not be used for the complete 3D reconstruction. Lymph nodes were frozen in Tissue-Tek O.C.T. Compound^TM^ at −80°C, cut into 16 μm thick slices using a cryostat (Leica CM3050 S) and stored at −80°C until they were washed, stained with DAPI for 5 min and covered with mounting medium.

### 3D Reconstruction

Each slice was put on a separate microscope slide and covered using (Dako Fluorescence Mounting Medium, Code S3023) mounting medium and a cover glass. The slides were then fully digitized using a digital slide scanner (Pannoramic Scan II, 3D HISTECH Ltd., Budapest, Hungary) at 20× magnification (pixel dimensions: 0.325 μm) and automatically stitched. The scanner is equipped with a quad band (DAPI/FITC/TRITC/Cy5) filter set, two filters (DAPI: nuclei for brain morphology, blue, 40 ms exposure time; FITC: fluorescent marker, green, 20 ms exposure time) were used for image acquisition. Due to the thickness of the slides they were digitized in extended focus mode with 30 focus layers and an axial layer distance of 2 μm.

**FIGURE 2:**
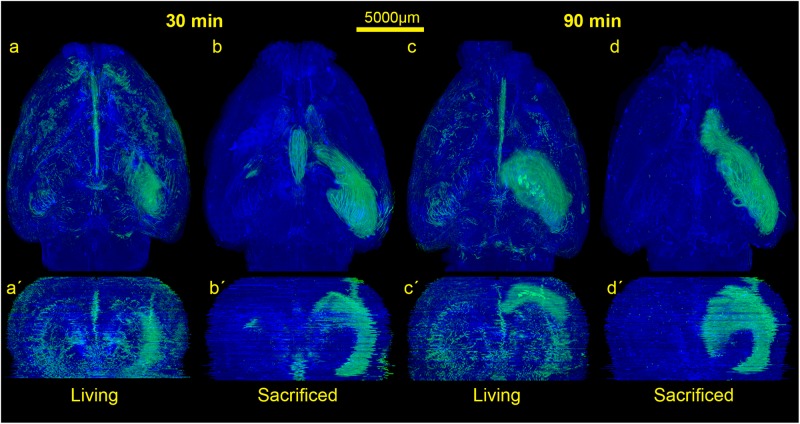
Comparison of the spread of the injected tracer in living and sacrificed rats at 30 min and 90 min after tracer injection. Images show maximum projections of horizontal sections **(a–d)** which were reconstructed coronally **(a’–d’)** of one animal per group. Evidently, the tracer reaches the contralateral hemisphere, spreads along the vasculature, fiber tracts and disseminates further within both hemispheres in living animals, while it diffuses mostly along ipsilateral hippocampal fiber tracts surrounding the ventricles in non-vital brains.

**FIGURE 3:**
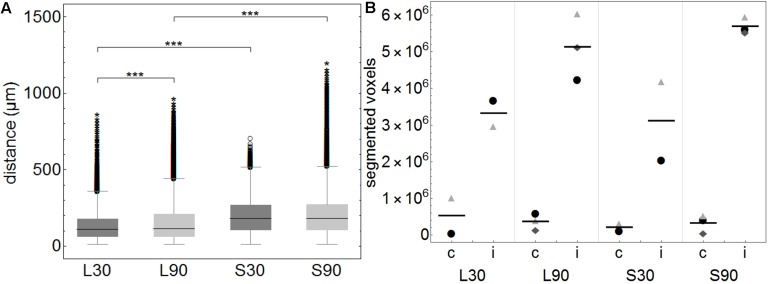
**(A)** Boxplots of calculated distances of every none-positive voxel within the brains to the closest positive voxel. Distance values of all 3D reconstructed individual brains (approximately 3450 voxels per brain) were summarized according to the groups L30 = living animals, 30 min PI, *n* = 2; L90 = living animals 90 min PI, *n* = 3; S30 = sacrificed animals 30 min PI, *n* = 2; S90 = sacrificed 90 min PI, *n* = 3. Shorter distances equal higher tracer spread. When comparing living to sacrificed groups tracer spread is significantly higher in living animals at both time points (***p ≤ 0.001). Tracer spread is significantly higher 30 min PI compared to 90 min PI in living animals, while there is statistically significant difference in sacrificed animals over time. **(B)** This subfigure shows the total number of segmented voxels per hemisphere. Within each group, identical symbols represent the corresponding hemispheres of one brain. A line is depicting the mean value. “c” the contralateral and “i” indicates the ipsilateral hemisphere of the injection site. Due to the very small group sizes a reliable statistical analysis could not be performed. Nevertheless, a trend of higher spread to the contralateral hemisphere in living animals is visible.

Images containing whole brain slices were exported from slide scanner data sets (Pannoramic Viewer, Version 1.15.4, 3D HISTECH Ldt., Budapest, Hungary) as PNG images with pixel dimensions of 10.4 μm. Subsequently, all images were coarsely cropped and aligned by hand (Adobe Photoshop CS6, Adobe Systems Inc., San Jose, CA, United States). The cerebellum was excluded due to massive artifacts from slide mounting. Slice images of each brain were converted to image stacks and underwent rigid registration using the “StackReg” plugin for ImageJ (Version 1.51n^1^). Only rigid registration was performed since results of elastic registration were heavily influenced by occurring tissue deformations/overlaps and missing or severed tissue portions. Further image analysis was performed with Mathematica (Version 11.1, Wolfram Research, Inc., Champaign, IL, United States). Registered image stacks were transformed into volumetric data sets and maximum projections along coronal, sagittal and horizontal axes were generated (Figure 2). Injection site was identified and marked within these projections, a separation line between the two hemispheres was also drawn (Adobe Photoshop CS6). Subsequently, all volumetric data sets were split into blue and green channels. Image slices of the blue channels were adjusted for brightness to retain consistent morphology. The green channel was binarized to detect tracer positive voxels. Binarized green channels were subsequently submitted to distance analysis. For this purpose, the distance of every non-positive voxel within the brains to the closest positive voxel was calculated with an average of 3450 voxel per brain. Distance values of individual brains were summarized according to the groups L30, L90, S30, and S90 (Figure 3). Shorter distances equal higher tracer spread. Distance values of all 10 3D reconstructed brains were shown as boxplots (Figure 3A). Based on the manually drawn separation lines the volumetric data sets of the green channels were split into ipsi- and contralateral hemispheres and all positive voxels were counted and plotted in a point chart (Figure 3B). Statistical analysis was performed using Mathematica. Shapiro Wilk test for normal distribution and Wilcoxon Signed Rank test for paired data were used to test data from the segmented voxel count analysis. Kolmogorov Smirnov test for normal distribution and Kruskal Wallis test for unpaired data were applied to test data from distance analysis. Dunn-Bonferroni *post hoc* correction after multiple pair comparisons was performed. *P*-values less than 0.05 were regarded as statistically significant. Volumetric data sets were visualized and animated (Supplementary Movies S1–S4) using Vaa3d (3D Visualization-Assisted Analysis, Version 3.20^2^). Injection site was interpolated and highlighted in the red image channel based on the marked position, and transparency of all three-color channels was adapted for best viewing experience.

Lymph node slides were also fully digitized using a digital slide scanner at 20× magnification following the procedure mentioned above except for an additional red channel to detect autofluorescent material. Exposure times were 100 ms for DAPI (lymph node morphology, blue), 150 ms for FITC (fluorescent marker, green) and 70 ms for TRITC (autofluorescent material, red). Images containing lymph nodes were exported from slide scanner data sets (Pannoramic Viewer, Version 1.15.4, 3D HISTECH Ldt., Budapest, Hungary) as PNG images with pixel dimensions of 0.65 μm.

Image analysis was performed with Mathematica. Fluorescence images were imported and split into separate color channels. After brightness equalization the red (autofluorescent material) channel was subtracted from the green (marker) channel to obtain autofluorescence corrected images. The remaining green channel was subsequently binarized using Otsu’s (cluster variance maximization) thresholding method (Otsu, 1979) and the number of segmented pixels representing detected fluorescence marker were counted. Statistical analysis was performed using Mathematica and boxplots were generated. Kolmogorov Smirnov test was applied to test data for normal distribution and Kruskal Wallis test with subsequent Dunn-Bonferroni *post hoc* correction after multiple pair comparisons was performed. *P*-values less than 0.05 were regarded as statistically significant.

### Immunofluorescent Stainings

After performing the 3D scanning for the 3D reconstruction, 20 representative brain sections were selected, uncovered and blocked in 3% DKS, 0.8% Triton, and 96.2% PBS. After washing with PBS, slides were covered with rabbit anti laminin antibody (Polyclonal rabbit Anti laminin; LOT: 00051761; ON: Z0097 Dako) diluted 1:200 and goat anti collagen IV (GtX Collagen Type IV; LOT: 2716603; ON: AB 769 Millipore) diluted 1:100 in 0.8% Triton, 0.5% DKS and 97.2% PBS overnight. The next day the slides were stained for 1 h with Alexa 647 donkey anti rabbit (Alexa Fluor 647 donkey anti rabbit; LOT: 1786284; ON: A31573 life technologies) (1:1 in Glycerol) and Alexa 568 donkey anti goat (Alexa Fluor 568 donkey anti goat IgG; LOT: 1235787; ON: A11057 life technologies) (1:1 in Glycerol) each diluted 1:250 in 0.08% Triton, 0.5% DKS and 97.1% PBS. Before covering the slides with mounting medium, they were stained for 5 min in DAPI.

Images of vessels and surrounding tissue close to as well as distant from the injection site were taken using Olympus microscope (Olympus BX 40) with a digital camera (Olympus XM10) At higher magnifications focus stacking of maximum 2 z-stacks per slide was used for better viewing experience. Images were manually masked (vessels, surrounding tissue, artifacts) using Photoshop. Subsequently, original images and manually generated masks were imported into Mathematica and intensity values for vessels and surrounding tissue regions were extracted. Total intensity for the two regions was added up and normalized by dividing the total intensity value by the area of the respective regions. Statistical analysis was performed using Mathematica and box plots were generated. Shapiro Wilk was applied to test data for normal distribution and *T*-test for paired data was used for group comparisons. *P*-values less than 0.05 were regarded as statistically significant.

## Results

In order to visualize the contribution of vital functions to the intraparenchymal spread of fluids, we injected 3 μl of a saturated solution of the fluorescein-coupled dextran amine Fluoro-Emerald into the entorhinal cortex of living and sacrificed rats and removed the brains 30 and 90 min afterward (*n* = 7 for 30 min per group and *n* = 3 for 90 min per group). The experimental setup is shown in Figure 1. Images of consecutive whole horizontal sections were taken at 20 times magnification and reconstructed three-dimensionally. Evidently, the tracer was transported along the vasculature and fiber tracts to remote areas including the contralateral hemisphere in living rats, while it appeared to diffuse along the ventricles and hippocampal fiber tracts in sacrificed animals, both at 30 and 90 min after injection (Figure 2 and Supplementary Movies S1–S4).

This is highlighted in the calculated 3D reconstructions where fluorescent voxels can be seen throughout both hemispheres. For each volumetric data set distance values of every non-positive voxel to the next positive voxel were calculated and summarized according to the groups L30, L90, S30, and S90 (Figure 3A). Shorter distances equal higher tracer spread. Kolmogorov Smirnov test revealed non-normal distribution of data from distance analysis. Kruskal Wallis test shows a highly significant increased tracer spread in living compared to sacrificed animals (*p* ≤ 0.001; with approximately 3450 voxels per brain) (Figure 3A). Furthermore, there is a significantly higher spread in living animals compared to sacrificed at 30 (110 vs. 181 μm) and 90 min (114 vs. 180 μm) PI (*p* ≤ 0.001). There is also a slightly higher spread in living animals 30 min (110 μm) PI compared to 90 min (114 μm) PI (*p* ≤ 0.001) (Figure 3A).

Quantification of the tracer distribution shows a notable spread to the contralateral hemispheres of the injection site in all animals (Figure 3B). In living animals spread to the contralateral hemisphere seems higher compared to sacrificed ones. No statistically significant difference was found when comparing this rough division using a relatively small number of animals (Figure 3B).

The rotating volumetric reconstructions exhibit the strong difference in tracer spread in both hemispheres between living and sacrificed brains for both time points. The 3D reconstructions make it more apparent that the tracer largely remains close to the injection site in the sacrificed brain. In contrast, the tracer in the living brain spreads along the fiber tracts such as the hippocampal fimbria/fornix complex (Supplementary Figure S1) and the vasculature to remote areas further away from the injection site. For better orientation the approximate injection site has been marked (Supplementary Movies S1–S4).

**FIGURE 4:**
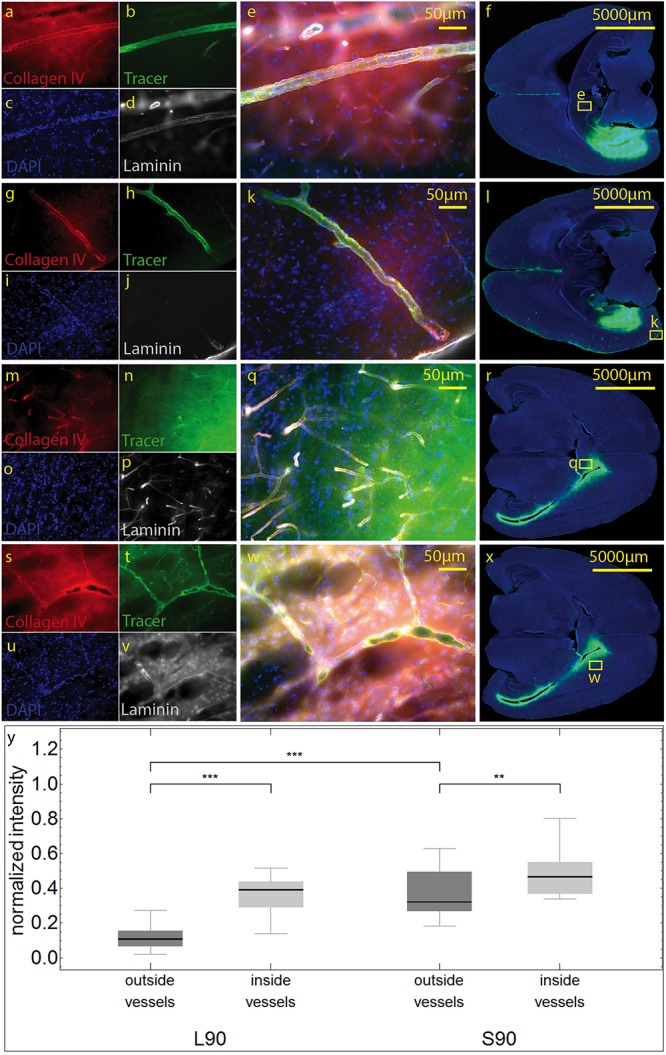
Stained sections of living **(a–l)** rats and a sacrificed rat **(m–x)** 90 min post injection. Representative sections were stained with antibodies against collagen IV **(a,g,m,s)**, laminin **(d,j,p,v)**, DAPI **(c,I,o,u)**, tracer **(b,h,n,t)**, magnification 20× **(a–e,g–k, m–q,s–w)**. The corresponding scanned horizontal section at magnification 0.2× provides the exact location of the taken pictures **(f,l,r,w)**. In living animals the tracer is clearly transported along the vascular wall not diffusing through the outer lamina. These vessels can be identified as arterioles by their size and the orientation of the two layers of nuclei. In sacrificed animals some of the tracer spreads along the vascular wall, but also provides a background staining of the neuropil **(m–q)**. The tracer accumulates in the parenchyma surrounding vessels/the perivascular space. **(s–w)**. Subfigure **(y)** shows normalized tracer intensity inside and outside of vessels. There is a highly significant difference for L90 (***p ≤ 0.001) and significant difference for S90 (**p = 0.003). The tracer amount outside of vessels in living animals is highly significant lower compared to sacrificed ones.

**FIGURE 5:**
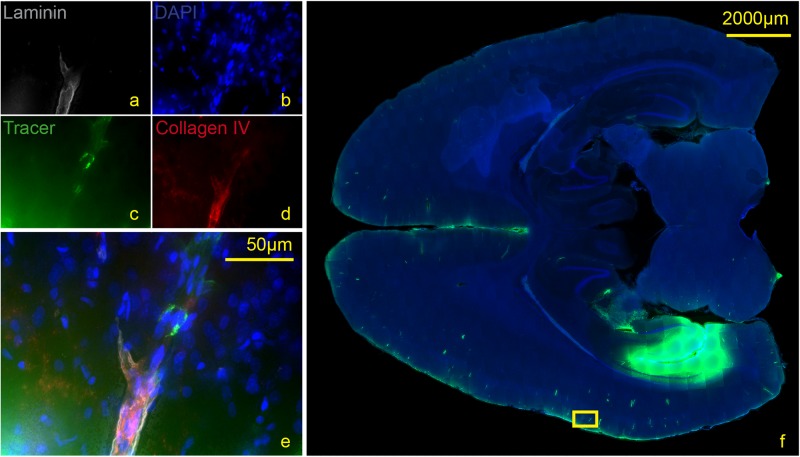
Stained section of a living rat 90 min post injection with antibodies against collagen IV **(d)**, laminin **(a)**, DAPI **(b)**, tracer **(c)**, magnification 40× **(a–e)**. The corresponding scanned horizontal section at magnification 0.2× provides the exact location of the taken picture **(f)**. Tracer accumulates within the inner and outer basement membrane ipsilateral to the injection site.

**FIGURE 6:**
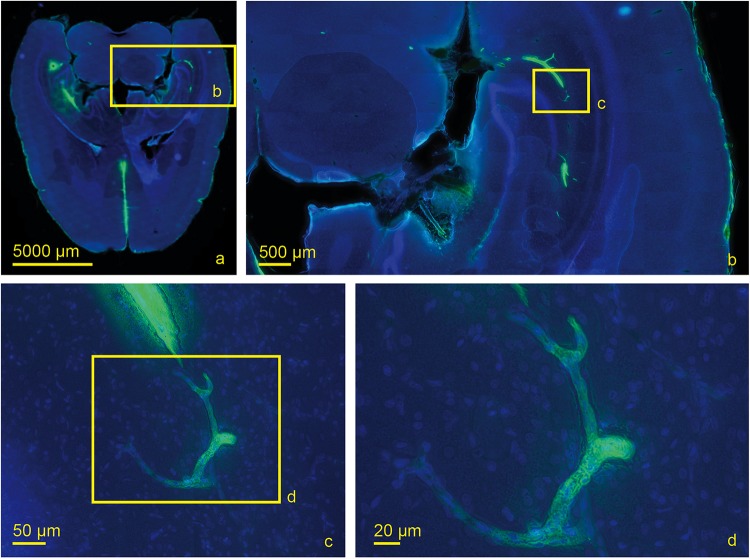
Scanned horizontal section of a living rat 30 min post injection with DAPI and tracer with increasing magnifications **(a)** 0.2×, **(b)** 2.0×, **(c)** 20×, **(d)** 40×. Tracer accumulation in the contralateral hemisphere occurs at the level of capillaries which are identified by their size.

We then used antibodies against collagen type IV and laminin to label the basement membranes of the neurovascular unit (Sixt et al., 2001; Bechmann et al., 2007; Engelhardt et al., 2016) and investigated the localization of the tracer within the vascular wall in sections counterstained with DAPI. Except for the immediate injection site where tissue was damaged, the tracer did not spread into the surrounding parenchyma (Figures 4m–r). In sacrificed animals the tracer was mainly located around the injection site. It spread within the closest ventricles as well as near fiber tracts, mainly remaining in the parenchyma, and reached the abluminal/external side of the vascular wall (Figures 4r–y). Shapiro Wilk test shows normal distribution. The *T*-test for paired data was performed and showed a highly significant difference for L90 (*p* ≤ 0.001) and significant difference for S90 (*p* = 0.003). The tracer amount outside of vessels in living animals is highly significant lower compared to sacrificed ones (*p* ≤ 0.001). Mean differences between intensity inside and outside of vessels was higher for living animals (L90 = 0.251) than for sacrificed animals (S90 = 0.114) (Figure 4y). In living animals, the tracer was found in the vascular outer and inner basement membrane (Figure 5). Tracer is also found along capillaries in the hemisphere contralateral to the injection site in living animals (Figure 6).

**FIGURE 7:**
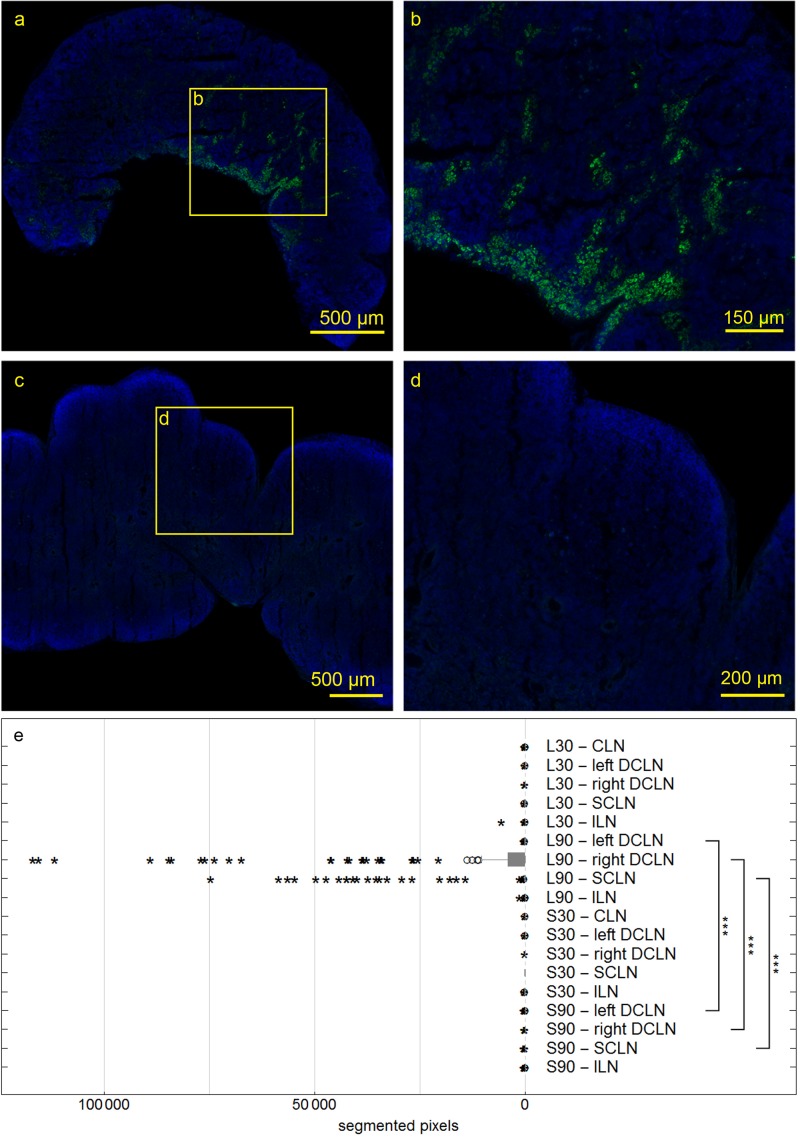
Deep cervical lymph nodes (DCLN) of rats 90 min post injection. Autofluorescent material was eliminated by subtracting the red channel from the pictures. In DCLN **(a,b)** of a living rat tracer is found within cells along the medullary trabecula, while no tracer could be detected within any dissected lymph node of sacrificed rats **(c,d)**. Box plot shows numbers of segmented voxels depicting fluorescent tracer within LN groups **(e)**. Tracer amounts in living compared to sacrificed animals 90 min post injection are significantly higher in right and left DCLN as well as SCLN (all ***p ≤ 0.001).

At 30 and 90 min, we also studied right and left deep cervical, superficial cervical and ILN, and searched for the tracer within them. In sacrificed rats, no tracer could be detected. However, at 90 min after tracer injection into living animals, ipsilateral deep cervical and SCLN nodes exhibited tracer signals in cells at the marginal and intermediate sinus (Figure 7a). Mann-Whitney test for paired data shows significantly higher tracer levels in living compared to sacrificed animals 90 min PI in right and left DCLN as well as SCLN (all *p* ≤ 0.001) with no difference for ILN and all lymph nodes 30 min PI.

## Discussion

In this study, we aimed at visualizing the impact of vital functions on the spread of a hydrophilic tracer within the brain’s neuropil. To this end, we compared maximum projections of three-dimensionally reconstructed brains from living and sacrificed rats 30 and 90 min after tracer injection into the entorhinal cortex. Our data show that the spread of extracellular fluids indeed is clearly enhanced in the presence of vital functions, suggesting that in living animals a fluid transport system contributes to tracer spread along the perivascular channel system. In contrast, in sacrificed animals diffusion mainly occurs either transparenchymal or across the vascular wall.

We found a highly significant increased tracer spread in living animals compared to sacrificed animals indicated by shorter distances between tracer positive and non-positive voxels in the volumetric reconstructions of the brain (Figures 3A,C). This includes perimortem changes which have been shown to play a crucial role in tracer spread (Ma et al., 2019). In the mentioned study tracer spread is detected in PVS of surface blood vessels after tracer injection into the lateral ventricle through the intact skull while life imaging, with an increase in perivascular tracer spread when the animal is sacrificed. The authors conclude that tracer spread to penetrating blood vessels only occurs under these conditions. We agree that perimortem changes/artifacts may contribute to the pattern of perivascular spread. With the shown top view and limited low resolution, it is not clear to us that penetrating blood vessels are excluded from tracer spread in living conditions. The infused tracer reaches the contralateral hemisphere and spreads along the vasculature and along fiber tracts in living animals, while it diffuses mostly along the ventricles and hippocampal fiber tracts in non-vital brains with less dissemination compared to living animals (Figures 2, 3B). Tracer spread is significantly lower in living animals 90 min compared to 30 min post injection (Figures 2, 3B). This could be explained by continues tracer clearance toward the cervical lymph nodes (Figure 7). At the moment it is unclear whether tracer penetrates into the ventricles or spreads along the subependymal space. Furthermore, the contribution of the changing perfusion pressure after death needs to be considered. In the sacrificed group it begins approximately 20 min before tracer injection. Since in both groups, the brains were immersion fixed, changing perfusion pressure before fixation occurs in both until the distribution of the PFA is complete. Tracer spread along fiber tracts has been described previously (Cserr et al., 1977; Rosenberg et al., 1980; Geer and Grossman, 1997). In contrast to the findings from Geer-Grossmann where frontal and temporal lobe injections were performed and the tracer remained within the same hemisphere, our study shows that dissemination of the tracer also occurs along the vasculature and fiber tracts in the contralateral hemisphere (Figures 2–6, Supplementary Movies S1–S4, and Supplementary Figure S1). Possible explanations could be faster needle removement after the injection, shorter infusion time, different injection sites, different tracers used or better tracer detection in our study. Cserr et al. (1977) used 0.5 μl whereas Geer-Grossmann infused 20 μl compared to 3 μl in our study. Thus, artifacts due to overflow must be considered but are difficult to predict and identify. Especially, as they would occur in both groups, living and sacrificed. In counterstained sections of living rats with laminin and collagen IV, the tracer is transported along the vascular wall of arterioles (Figures 4a–l, 5 and Supplementary Figure S2) and capillaries (Figure 6) as described previously (Carare et al., 2008; Morris et al., 2016) not spreading into the surrounding parenchyma, except for the immediate injection site where a degree of brain damage occurred. Possible leakage to the peripheral circulation cannot be excluded but seems to play a minor role since tracer spread has not occured in the shape of venous drainage. Contradicting, in sacrificed animals tracer is continuously detected after transparenchymal or transvascular diffusion. A limiting fact is that in the present study, vessels were only identified as arterioles by their size and the orientation of the two layers of nuclei. Similar studies using detailed characterization of the distribution of tracers demonstrate that they enter the parenchyma along arterial pial-glial basement membranes and tracers within the parenchyma enter the basement membranes surrounding smooth muscle cells, as IPD (Morris et al., 2016; Albargothy et al., 2018). In sacrificed rats the tracer was also observed to spread along fiber tracts and to accumulate within vessel walls, but the driving force is unclear at present. Non-physiological changes in vital processes concerning the cessation of the sacrificed group as well as postmortem changes need to be considered besides diffusion. It certainly would be interesting to perform further experiments comparing intracisternally applied tracer in living vs. sacrificed vs. sacrificed immediately after tracer injection to study the perimortem changes in greater detail. Furthermore, intraparenchymal injections in animals that were sacrificed after the stereotactic placement of the syringe right before tracer injection would help to reduce postmortem artifacts in the sacrificed group. To avoid blood clotting in the sacrificed group, transcardial perfusion with heparinized animals would minimize artifacts from the immersion fixation. Another possible limitation is the use of a saturated tracer solution, which might interfere with physiological processes differently than a more diluted tracer, as well as the intraparenchymal injected amount of 3 μl which may lead to localized pressure changes and therefore possibly affects tracer distribution in sacrificed and living animals differently. Corresponding with previous findings (Bradbury et al., 1981; Szentistványi et al., 1984; Ma et al., 2019) tracer is found within deep cervical and SCLN, specifically within cells along the medullary trabecular and not in ILN or sacrificed animals after intracerebral injection. The tracer could microscopically not be displayed in contralateral DCLN. This discrepancy could for one be explained with the shorter time points after intracerebral injection (90 min vs. 5–7 h), meaning that tracer did not yet reach the contralateral side in our study. Additionally, different animals were used (mice (Ma et al., 2019) vs. rabbit (Bradbury et al., 1981) vs. rat), different injections sites (intracisternal (Ma et al., 2019) vs. intraparenchymal (Bradbury et al., 1981) and our study), different tracer sizes 10kDa (our study) vs. 40 kDa (Ma et al., 2019) and different imaging techniques. Recognizing the previously described drainage via lymphatic vessels in dural sinuses (Louveau et al., 2015) in the present study we did not analyze the sagittal sinus for tracer spread. Statistical analysis of the highly sensitive image segmentation showed significantly higher tracer levels in living compared to sacrificed animals 90 min post injection in right and left DCLN as well as SCLN even though microscopically no tracer could be seen contralaterally. The tracer may have begun to appear contralateral to the injection side but has not shown the same distribution pattern as in ipsilateral LN yet. In agreement with former findings (Bradbury et al., 1981; Koh et al., 2005; Ma et al., 2017, 2019), where intracisternally and intraventricular applied tracer first reached cervical lymph nodes and then concentration increased in the blood, we hypothesize that clearance from the brain and extracellular fluid occurred via ISF and CSF into cervical lymph nodes in living animals. We cannot exclude spillage into the CSF compartment of our tracer, although we took every precaution possible to prevent this.

Further studies are needed to characterize the tracer-fluorescent cells at the marginal and intermediate sinus to investigate if they phagocytosed the tracer after it reached the lymph nodes, if they internalized tracer before (e.g., intracranially and then drained to the lymph nodes) or if the tracer only adhered to the surface of these cells.

## Conclusion

The spread of extracellular fluids depends on and increases in the presence of vital functions. In vital brains an intraparenchymal injected tracer is transported along the vasculature and fiber tracts to remote areas including the contralateral hemisphere. The tracer remained within the wall of the vasculature, almost solely found within the outer and inner basement membrane of the vessels, not spreading into the surrounding parenchyma. In contrast, in sacrificed animals, the tracer spread along ventricles and fiber tracts (predominantly the fornix), aligned with the outer vascular border close to the injection site. Thus, the presence of an active clearance system driven by vital functions is supported by the different patterns of distribution of the tracers in living and sacrificed animals. Furthermore, the intracerebrally injected tracer was only found in the cervical lymph nodes of living animals, suggesting that the drainage of ISF/CSF to the cervical lymph nodes is an active process.

## Data Availability Statement

The datasets used and analyzed during the current study are available from the corresponding author on reasonable request.

## Ethics Statement

The animal study was reviewed and approved by the Landesdirektion Sachsen, Germany; TV03/13.

## Author Contributions

AP performed the experiment, tissue processing, histological examination of the slides and prepared the scanned sections for further processing, writing the manuscript. KW digitized all microscopic slides, performed all image processing (3D reconstructions, maximum projections, segmentation of 2D/3D image data, etc.) and statistical analysis. IB supervised the project, was involved in the conception and design. AP and IB were the major contributors in writing the manuscript. RC revised it critically for intellectual content. All authors read and approved the final manuscript.

## Conflict of Interest

The authors declare that the research was conducted in the absence of any commercial or financial relationships that could be construed as a potential conflict of interest.

## Footnotes

1 http://imagej.nih.gov/ij

2 http://vaa3d.org

## Abbreviations

° C: degree Celsius
μ l: microliter
μ m: micrometer
3D: three-dimensional
AQP4: aquaporin 4
CSF: cerebrospinal fluid
DAPI: 4 ′6-Diamidin-2-phenylindol
DCLN: deep cervical lymph nodes
DKS: donkey serum
GAD: glutaraldehyde
ICP: intracranial pressure
IgG: immunoglobulin G
ILN: inguinal lymph nodes
IPAD: intramural periarterial drainage
ISF: interstitial fluid
kDa: kilodalton
min: minute
mm: millimeter
mM: millimolar
ms: millisecond
NaCl: sodium chloride
PBS: phosphate buffered saline
PFA: paraformaldehyde
PI: post injection
PVS: perivascular space
SCLN: superficial cervical lymph nodes.
