# Supplementary figures and images for: Vital Functions Contribute to the Spread of Extracellular Fluids in the Brain: Comparison Between Life and Death
Source: Front Aging Neurosci. 2020 Feb 11;12:15. doi: 10.3389/fnagi.2020.00015 (PMC7027336; doi:10.3389/fnagi.2020.00015)

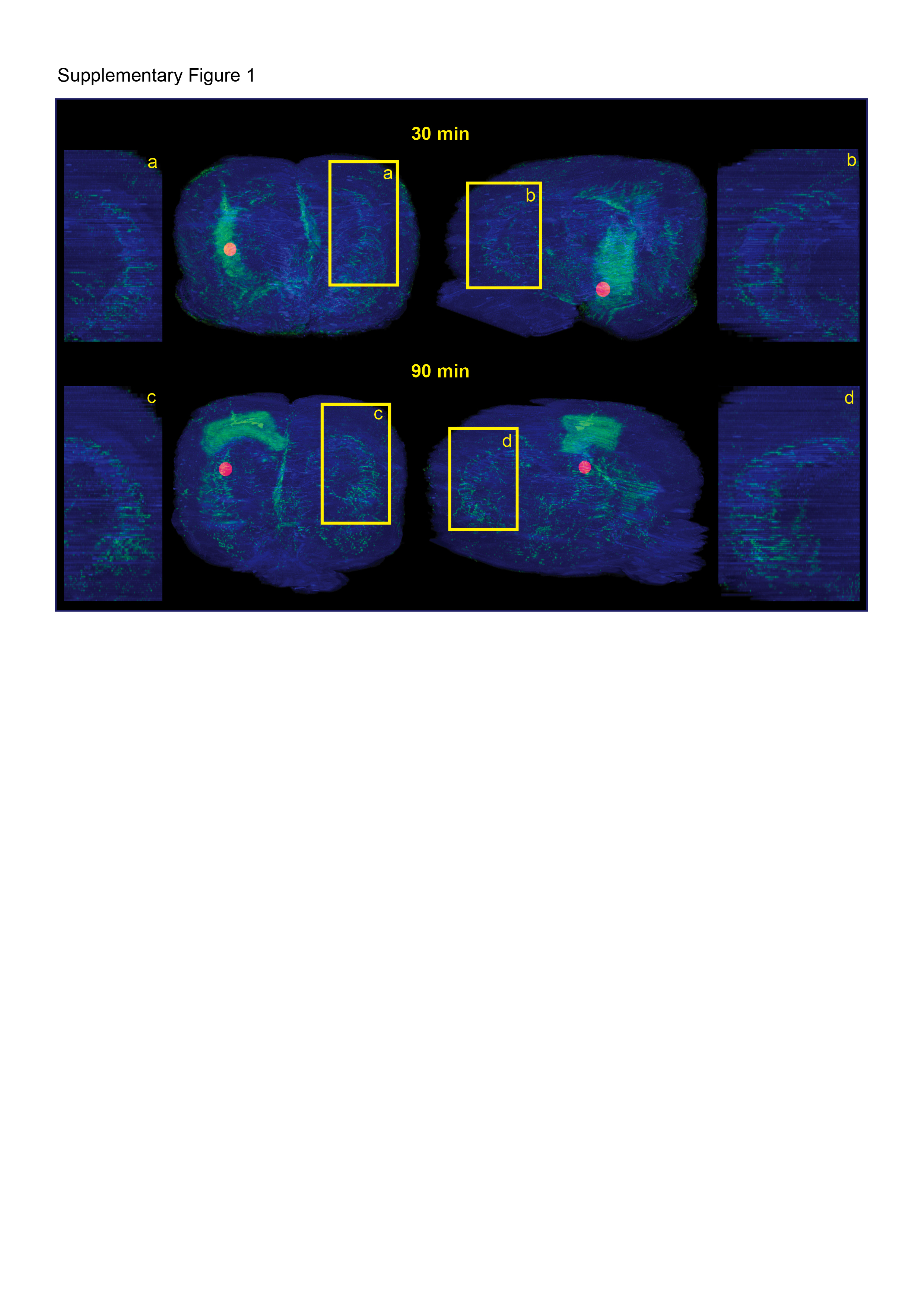

Supplement: Supplementary file 1 [file Image_1.JPEG]

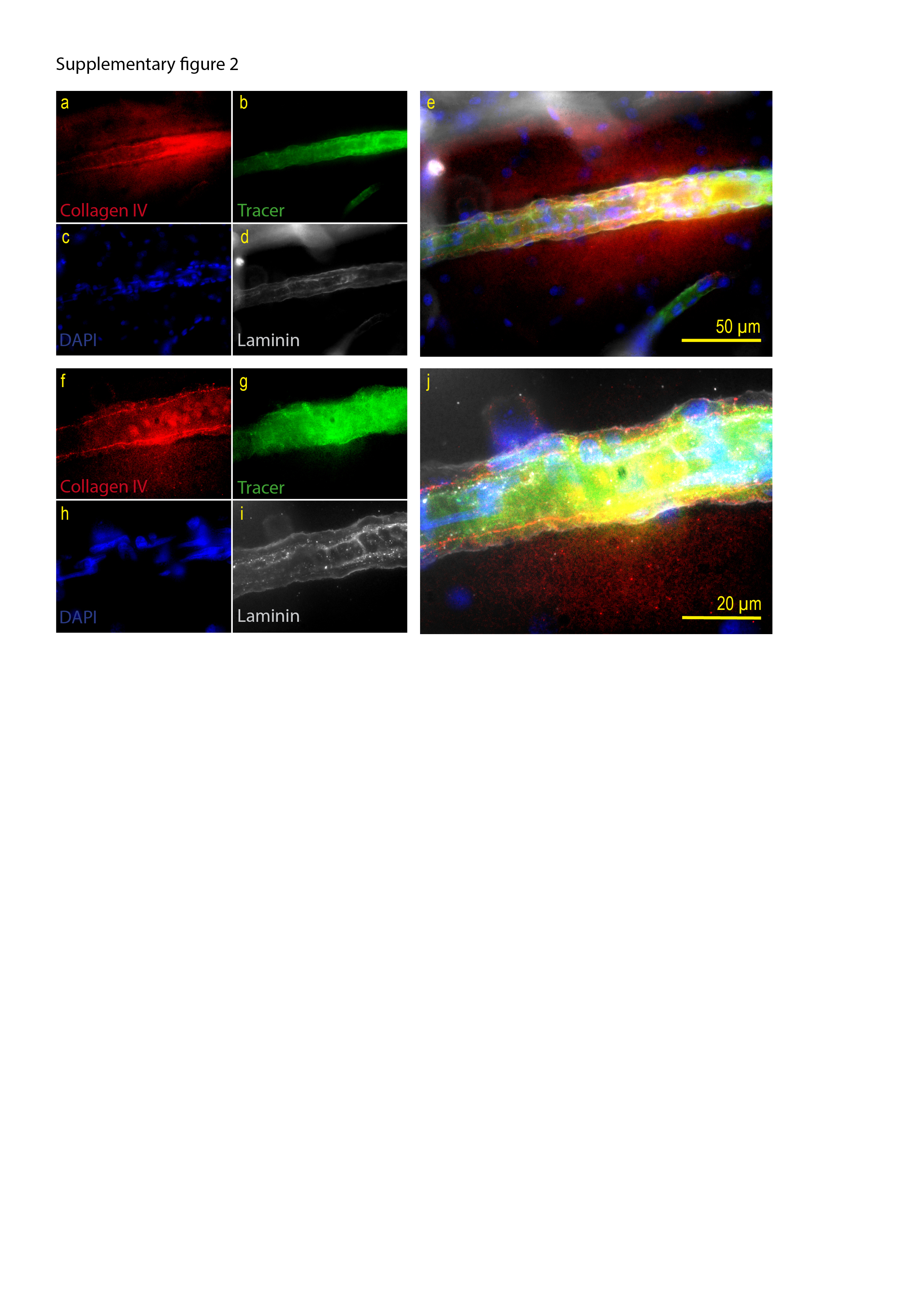

Supplement: Supplementary file 2 [file Image_2.JPEG]
