# Supplementary material for: Vital Functions Contribute to the Spread of Extracellular Fluids in the Brain: Comparison Between Life and Death
Source: Front Aging Neurosci. 2020 Feb 11;12:15. doi: 10.3389/fnagi.2020.00015 (PMC7027336; doi:10.3389/fnagi.2020.00015)
Supplement: Supplementary file 7 [file Table_1.docx]

Supplementary Material

# Movie 1-4

Rotating animation of the 3D reconstructed brains of a living rat 30 min post injection (PI) (1), sacrificed animal 30 min PI (2), living rat 90 min PI (3) and sacrificed animal 90 min PI (4). The approximate injection site has been marked. The tracer remains largely close to the injection site in the sacrificed brains and disseminates mainly around the ipsilateral lateral ventricle (2, 4), whereas it spreads along the fiber tracts and vasculature to remote areas away from the injection site (1,3).

# Supplementary figure 1

Enlarged view of the 3D reconstructed hippocampal fimbria/fornix complex contralateral to the injection site of living animals 30 min (a, b) and 90 min (c, d) post injection. Tracer spread in the shape of the fornix can be appreciated at both time points.

# Supplementary figure 2

Stained sections of living rats 90 min post injection represent higher magnification of figure 4e. Sections were stained with antibodies against collagen IV (a, f), laminin (d, i), DAPI (c, h), tracer (b, g), magnification 40x (a - e), 100x (f - j). The tracer is transported along the vascular wall not diffusing through the outer lamina. These vessels can be identified as arterioles by their size and the orientation of the two layers of nuclei.
